# Solitary lymph node metastasis is a distinct subset of colon cancer associated with good survival: a retrospective study of surveillance, epidemiology, and end-results population-based data

**DOI:** 10.1186/1471-2407-14-368

**Published:** 2014-05-24

**Authors:** Qingguo Li, Yuwei Wang, Guoxiang Cai, Dawei Li, Sanjun Cai

**Affiliations:** 1 Department of Colorectal Surgery, Fudan University Shanghai Cancer Center, Department of Oncology, Shanghai Medical College, Fudan University, 270 Dong’an Road, Shanghai 20032, China

**Keywords:** Colon Cancer, Lymph node metastasis, Surgery, Survival analysis

## Abstract

**Background:**

Colon cancer with lymph node metastases has been considered as advanced stage and to have poor survival. We postulated that patients with solitary lymph node metastasis are a distinct subset with better colon cancer-specific survival than those with multiple lymph node metastases.

**Methods:**

In this retrospective study, we searched Surveillance, Epidemiology, and End-Results (SEER) population-based data and identified 86,674 patients who had been diagnosed with colon cancer without distant metastases and with less than three metastatic nodes between 1991 and 2005. We divided lymph node status into three subgroups: pN0, pN1a, and pN1b and obtained 5-year colon cancer-specific survival for each pT stage. We used Kaplan–Meier and multivariate Cox regression models to assess correlations between risk factors and survival outcomes.

**Results:**

Analysis of SEER data confirmed that patients with solitary lymph node metastases had better 5-year cancer-specific survival than pN1b according to both univariate and multivariate analysis. This finding was confirmed by further analyses in five pT subgroups*.* Cancer-specific survival of patients with pT1-2N1a was comparable to that of those with pIIA but higher than those with pIIB. In addition, survival of patients with pT3-4aN1a was better than those with pIIC.

**Conclusion:**

Colon cancer patients with solitary lymph node metastasis are a distinct subset with a favorable prognosis; full consideration should be given to this in clinical practice.

## Background

Colorectal cancer (CRC), one of the commonest malignancies, is the third leading cause of cancer-related deaths in the United States [1]. The incidence of CRC in Asian countries is increasing rapidly and is likely similar to that in Western countries [2,3]. In China, both the incidence and mortality rate of CRC are increasing [4]. Surgical resection remains the mainstay of treatment of local and regional disease. Lymphadenectomy, a critical component of surgical procedures for patients with CRC, is performed with the aim of achieving complete resection of lesions. In 2000, the National Comprehensive Cancer Network (NCCN) recommended pathologic examination of at least 12 lymph nodes (LNs) in the staging of colon cancer (CC). The number of metastatic LNs has been identified as an independent prognostic factor [5-7]. In the seventh edition of the American Joint Committee on Cancer (AJCC) Cancer Staging Manual for CC, N1 lesions were subdivided into N1a (solitary LN metastasis, SLNM) and N1b (2–3 positive LNs); however, in the current staging system N1a and N1b have been combined. Patients with SLNM might be a distinct subset of those with involved LNs, a subset without the high incidence of systematic disease and poor prognosis of patients with multiple metastases in LNs. In this study, we used data from the Surveillance, Epidemiology and End-Results (SEER) registries to analyze the role of SLNM in the long-term survival of patients with CC and to assess the appropriateness of the N1 classification in the seventh edition of the TNM staging system.

## Methods

The current SEER database consists of 17 population-based cancer registries that represent approximately 28% of the population of the United States. The SEER data contain no identifiers and are publicly available for studies of cancer-based epidemiology and health policy. The National Cancer Institute’s SEER*Stat software (Surveillance Research Program, National Cancer Institute SEER*Stat software, http://www.seer.cancer.gov/seerstat) was used to identify patients who received a pathologic diagnosis of adenocarcinoma, mucinous adenocarcinoma, or signet-ring carcinoma of the CC (C18.0–19.9) between 1991 and 2005.

Only CC as a single primary tumor was included in current study due to the available information for cause specific survival analysis in SEER database. Patients diagnosed after 2006 were excluded to ensure adequate duration of follow-up. Other exclusion criteria were as follows: incomplete TNM staging, no LNs examined pathologically, more than three LNs with metastases (N2), synchronous distant metastases, patients who had died within 30 days of surgery, and age younger than 18 or older than 80 years.

This study is based on public data from the SEER database: we obtained permission to access the research data files in the SEER program (reference number 12768-Nov2012). Because this study did not involve interaction with human subjects or use personal identifying information, informed consent was not required. The study was approved by the Review Board of Fudan University, Shanghai Cancer Center, Shanghai, China.

### Ethics statement

This study was conducted in compliance with the Helsinki Declaration. Permission to access the research data files in the SEER program was obtained (reference number 12768-Nov2012).

### Statistical analysis

Age, sex, race, extent of primary tumor invasion, total number of LNs examined, number of involved LNs, tumor grade, histological type of tumor, survival time, and cause of death were retrieved from the SEER database. All cases were restaged based on the AJCC-7 guidelines. The primary endpoint of this study, colon cancer cause-specific survival (CCSS), was calculated from the date of diagnosis to the date of cause-specific death. Deaths attributed to the cancer of interest were treated as events and deaths from other causes as censored observations.

*χ*^2^ tests were used to test independence, and Student’s *t*-test to compare continuous data between the three groups (pN0, pN1a, and pN1b). Exact 95% confidence intervals (CIs) for proportions were calculated. Survival curves were generated using Kaplan–Meier estimates; differences between the curves were analyzed by the log-rank test. Multivariate Cox regression models were used to analyze correlations between risk factors and survival outcomes in T1-4 N0-1b patients. All statistical analyses were performed with the statistical software package SPSS for Windows, version 17 (SPSS, Chicago, IL, USA). Statistical significance was set at two-sided *P* < 0.05.

## Results

### Impact of SLNM on CC survival outcomes

We identified 86,674 eligible patients over the 15 years covered by the study. These comprised 61,696 patients with no LN metastases, 12,416 with SLNM, and 12,562 with two or three LN metastases. Relevant patient characteristics and pathological features are summarized in Table 1. LN status was correlated with age, race, pathological grading, histological type of tumor, number of LNs dissected, and pT stage.

**Table 1:** Characteristics of patients from SEER Database by LN involvement

	**Total**	**N0**	**N1a**	**N1b**	**P value**
**Characteristic**	**(n = 86674)**	**(n = 61696)**	**(n = 12416)**	**(n = 12562)**	
Media follow up (mo)	85	90	78	69	<0.001
(IQR)	(54–121)	(62–124)	(39–116)	(30–108)	
Years of diagnosis					0.102
1988-1993	17214	12196	2489	2529	
1994-1999	24641	17436	3629	3576	
2000-2003	32064	17436	6298	6457	
Sex					0.818
Male	43210	30798	6177	6235	
Female	43464	30898	6239	39876327	
Age					<0.001
<60	27442	18170	4293	4439	
≥60	59232	42986	8123	8123	
Race					<0.001
White	69850	50278	9667	9905	
Black	9407	6337	1574	1496	
Other	7154	4872	1145	1137	
Unknown	263	209	30	24	
Pathological grading					<0.001
High/moderate	41097	28840	6121	6136	
Poor/anaplastic	7723	4503	1434	1786	
Unknown	1750	1232	251	267	
Histotype					<0.001
Adenocarcinoma	76387	54697	10817	10873	
Mucinous cell	9667	6671	1480	1516	
Signet ring cell	486	239	96	151	
T stage					<0.001
T1	12141	11055	746	340	
T2	14570	11995	1531	1044	
T3	28192	18982	4532	4678	
T4a	27669	17195	4860	5614	
T4b	4102	2469	747	886	
No. of LNs dissected					<0.001
<12	47920	34671	6693	6556	
≥12	38754	27025	5723	6006	

**Figure 1:**
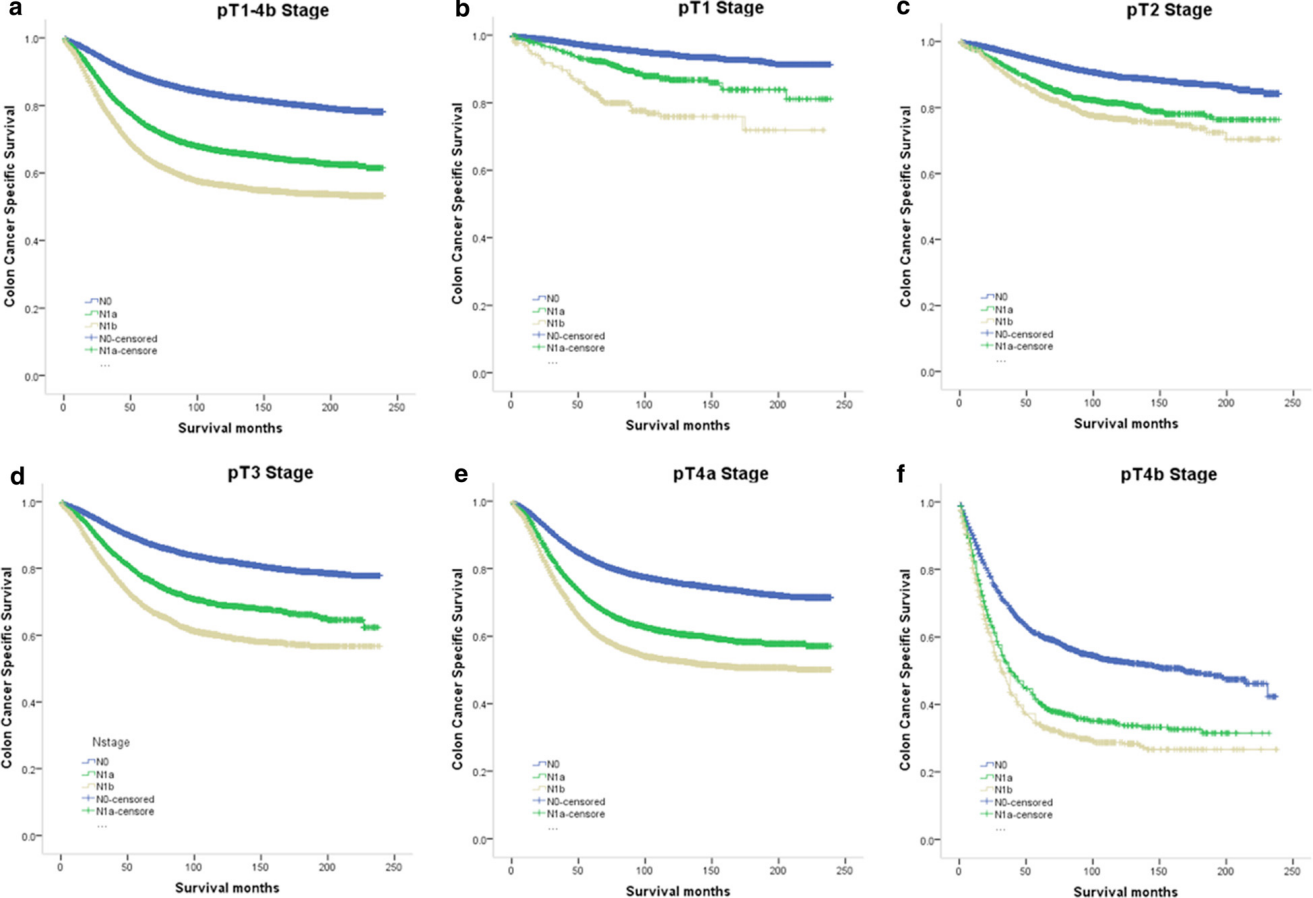
**Survival curves in CC patients according to lymph node status. (a)** pT1-4 stage N0 vs. N1a, *χ*^*2*^ *=* 1762.258*, P <* 0.001; N1a vs. N1b, *χ*^*2*^ *=* 263.886*, P <* 0.001*.***(b)** pT1 stage: N0 vs. N1a, *χ*^*2*^ *=* 53.979*, P <* 0.001; N1a vs. N1b, *χ*^*2*^ *=* 21.414*, P <* 0.001*.***(c)** pT2 stage: N0 vs. N1a, *χ*^*2*^ *=* 101.579*, P <* 0.001; N1a vs. N1b, *χ*^*2*^ *=* 5.597*, P =* 0.02*.***(d)** pT3 stage: N0 vs. N1a, *χ*^*2*^ *=* 374.208*, P <* 0.001; N1a vs. N1b, *χ*^*2*^ *=* 86.490*, P <* 0.001*.***(e)** pT4a stage: N0 vs. N1a, *χ*^*2*^ *=* 420.664*, P <* 0.001; N1a vs. N1b, *χ*^*2*^ *=* 71.364*, P <* 0.001*.***(f)** pT4b stage: N0 vs. N1a, *χ*^*2*^ *=* 94.180*, P <* 0.001; N1a vs. N1b, *χ*^*2*^ *=* 10.257*, P =* 0.001.

The median duration of follow-up was 85 months (range 54–121 months) and the overall 5-year CCSS was 83.0%. The 5-year CCSS of pN0 patients, patients with pN1a and patients with pN1b stage was 88.3% ± 0.1%, 74.6% ± 0.4%, and 65.1% ± 0.4%, respectively (*P* < 0.001). There were significant differences in survival between pN0 patients and those with SLNM (*P* < 0.001), between patients with SLNM and with pN1b (*P* < 0.001), and between patients with pN0 and pN1b *(P* < 0.001). We then made a further comparison by pT stages and found significant differences between all five of them (*P* < 0.05) (Figure 1).

According to univariate and multivariate survival analyses, pT stage, year of diagnosis, patient age, race, and LN status were significantly associated with CCSS in all patients. pT2-4a stage female patients had better CCSS than male patients. Tumor grade was an independent factor for CCSS in patients with pT1 and pT3-4b. Except in patients with pT1 stage, the number of LNs dissected was significantly associated with CCSS according to both univariate and multivariate survival analysis. However, histological type of tumor was not a prognostic factor according to both univariate and multivariate survival analyses (Tables 2, 3, 4, 5 and 6).

**Table 2:** Univariate and multivariate survival analyses by pN stage in patients with pT1 stage CC

		**Univariate analysis**		**Multivariate analysis**	
**Variable**	**5-year CCS**	**Log rank *χ***^**2 **^**test**	**P**	**HR (95% CI)**	**P**
Years of diagnosis		15.944	<0.001		<0.001
1988-1993	94.9%			Reference	
1994-1999	95.6%			0.815 (0.784-0.848)	
2000-2003	96.6%			0.692 (0.667-0.718)	
Sex		0.706	0.401		NI
Male	96.0%				
Female	96.2%				
Age		45.295	<0.001		<0.001
<60	97.6%			Reference	
≥60	95.3%			1.467 (1.419-1.516)	
Race		16.447	<0.001		<0.001
White	96.3%			Reference	
Black	93.4%			1.428 (1.152-1.770)	
Other^a^	97.6%			0.718 (0.528-0.976)	
Grade		19.124	<0.001		<0.001
High/moderate	96.2%			Reference	
Poor/anaplastic	93.1%			1.281 (1.234-1.330)	
Unknown	96.8%			0.871 (0.802-0.946)	
Histotype		0.923	0.337		NI
Adenocarcinoma	96.1%				
Mucinous/signet ring cell	96.8%				
No. of LNs dissected		0.413	0.520		NI
<12	95.9%				
≥12	96.5%				
LNs status		221.646	<0.001		<0.001
N0 (pI)	96.7%			0.456 (0.439-0.473)	
N1a	92.6%			Reference	
N1b	83.2%			1.424 (1.365-1.486)	

^a^Other includes American Indian/Alaska native, Asian/Pacific Islander, and unknown.

*NI*: not included in multivariate survival analyses.

**Table 3:** Univariate and multivariate survival analyses by pN stage in patients with pT2 stage CC

		**Univariate analysis**		**Multivariate analysis**	
**Variable**	**5-year CCS**	**Log rank *χ***^**2 **^**test**	**P**	**HR (95% CI)**	**P**
Years of diagnosis		30.763	<0.001		<0.001
1988-1993	90.9%			Reference	
1994-1999	92.0%			0.903 (0.789-1.033)	
2000-2003	93.6%			0.727 (0.637-0.830)	
Sex		27.577	<0.001		<0.001
Male	91.8%			Reference	
Female	93.5%			0.734 (0.664-0.812)	
Age		77.274	<0.001		<0.001
<60	95.4%			Reference	
≥60	91.5%			1.823 (1.612-2.061)	
Race		35.396	<0.001		<0.001
White	92.8%			Reference	
Black	89.6%			1.517 (1.306-1.762)	
Other^a^	94.9%			0.692 (0.558-0.858)	
Grade		4.629	0.099		NI
High/moderate	92.9%				
Poor/anaplastic	90.9%				
Unknown	91.4%				
Histotype		0.190	0.663		NI
Adenocarcinoma	92.9%				
Mucinous/signet ring cell	92.5%				
No. of LNs dissected		20.732	<0.001		<0.001
<12	91.7%			Reference	
≥12	94.0%			0.846 (0.761-0.941)	
LNs status		223.132	<0.001		0.002
N0	94.2%			0.485 (0.423-0.556)	
N1a	87.2%			Reference	
N1b	83.8%			1.270 (1.060-1.521)	

^a^Other includes American Indian/Alaska native, Asian/Pacific Islander, and unknown.

*NI*: not included in multivariate survival analyses.

**Table 4:** Univariate and multivariate survival analyses by pN stage in patients with pT3 stage CC

		**Univariate analysis**		**Multivariate analysis**	
**Variable**	**5-year CCS**	**Log rank *χ***^**2 **^**test**	**P**	**HR (95% CI)**	**P**
Years of diagnosis		39.995	<0.001		<0.001
1988-1993	80.3%			Reference	
1994-1999	82.6%			0.874 (0.808-0.946)	
2000-2003	84.6%			0.820 (0.763-0.881)	
Sex		25.387	<0.001		<0.001
Male	82.7%			Reference	
Female	84.3%			0.855 (0.811-0.901)	
Age		169.293	<0.001		<0.001
<60	87.3%			Reference	
≥60	81.7%			1.542 (1.453-1.638)	
Race		103.809	<0.001		<0.001
White	84.1%			Reference	
Black	77.7%			1.461 (1.355-1.574)	
Other^a^	86.2%			0.801 (0.733-0.896)	
Grade		15.823	<0.001		0.032
High/moderate	84.0%			Reference	
Poor/anaplastic	80.8%			1.098 (1.024-1.178)	
Unknown	83.5%			1.003 (0.851-1.182)	
Histotype		1.212	0.271		NI
Adenocarcinoma	83.5%				
Mucinous/signet ring cell	83.8%				
No. of LNs dissected		270.983	<0.001		<0.001
<12	80.0%			Reference	
≥12	86.8%			0.668 (0.633-0.705)	
LNs status		1209.713	<0.001		<0.001
N0 (pIIA)	88.4%			0.510 (0.476-0.546)	
N1a	77.8%			Reference	
N1b	69.4%			1.449 (1.334-1.561)	

^a^Other includes American Indian/Alaska native, Asian/Pacific Islander, and unknown.

*NI*: not included in multivariate survival analyses.

**Table 5:** Univariate and multivariate survival analyses by pN stage in patients with pT4a stage CC

		**Univariate analysis**		**Multivariate analysis**	
**Variable**	**5-year CCS**	**Log rank *χ***^**2 **^**test**	**P**	**HR (95% CI)**	**P**
Years of diagnosis		61.405	<0.001		<0.001
1988-1993	72.8%			Reference	
1994-1999	77.1%			0.836 (0.792-0.883)	
2000-2003	77.8%			0.848 (0.803-0.896)	
Sex		35.224	<0.001		<0.001
Male	75.1%			Reference	
Female	77.2%			0.859 (0.822-0.898)	
Age		136.610	<0.001		<0.001
<60	79.9%			Reference	
≥60	74.5%			1.383 (1.316-1.454)	
Race		85.397	<0.001		<0.001
White	76.7%			Reference	
Black	69.1%			1.357 (1.270-1.450)	
Other^a^	79.7%			0.845 (0.777-0.918)	
Grade		52.978	<0.001		0.032
High/moderate	77.3%			Reference	
Poor/anaplastic	71.5%			1.178 (1.116-1.244)	
Unknown	75.5%			1.053 (0.917-1.210)	
Histotype		0.011	0.915		NI
Adenocarcinoma	76.2%				
Mucinous/signet ring cell	76.0%				
No. of LNs dissected		266.370	<0.001		<0.001
<12	72.4%			Reference	
≥12	80.4%			0.708 (0.676-0.740)	
LNs status		1213.378	<0.001		<0.001
N0 (pIIB)	82.7%			0.559 (0.528-0.592)	
N1a	69.9%			Reference	
N1b	61.7%			1.317 (1.239-1.401)	

^a^Other includes American Indian/Alaska native, Asian/Pacific Islander, and unknown.

*NI*: not included in multivariate survival analyses.

**Table 6:** Univariate and multivariate survival analyses by pN stage in patients with pT4b stage CC

		**Univariate analysis**		**Multivariate analysis**	
**Variable**	**5-year CCS**	**Log rank *χ***^**2 **^**test**	**P**	**HR (95% CI)**	**P**
Years of diagnosis		37.575	<0.001		0.001
1988-1993	44.4%			Reference	
1994-1999	49.2%			0.905 (0.807-1.014)	
2000-2003	55.7%			0.809 (0.726-0.902)	
Sex		1.163	0.281		NI
Male	51.9%				
Female	50.7%				
Age		41.821	<0.001		<0.001
<60	56.7%			Reference	
≥60	48.0%			1.329 (1.213-1.456)	
Race		19.460	<0.001		<0.001
White	52.0%			Reference	
Black	42.3%			1.337 (1.175-1.522)	
Other^a^	56.4%			0.920 (0.781-1.084)	
Grade		48.208	<0.001		<0.001
High/moderate	54.3%			Reference	
Poor/anaplastic	44.8%			1.343 (1.219-1.479)	
Unknown	41.7%			1.324 (1.094-1.601)	
Histotype		0.024	0.877		NI
Adenocarcinoma	51.2%				
Mucinous/signet ring cell	51.4%				
No. of LNs dissected		158.496	<0.001		<0.001
<12	42.4%			Reference	
≥12	60.4%			0.598 (0.548-0.653)	
LNs status		1213.378	<0.001		<0.001
N0 (pIIC)	60.6%			0.596 (0.534-0.666)	
N1a	40.5%			Reference	
N1b	34.1%			1.201 (1.062-1.358)	

^a^Other includes American Indian/Alaska native, Asian/Pacific Islander, and unknown.

NI: not included in multivariate survival analyses.

### Comparison of CCSS between patients with pT1-4aN1a and those with pII stage CC

As presented in Tables 2, 3, 4, 5 and 6, the 5-year CCSS of patients with pIIA, pIIB, and pIIC CC were 88.40%, 82.70%, and 60.60%, respectively, all being lower than that of those with pT1N1a (92.60%). The 5-year CCSS of patients with pIIB and pIIC CC was lower than that of those with pT2N1a (87.20%) and that of patients with pIIC lower than that of those with pT3N1a (69.90%). According to AJCC-7 T classification in stage III, we made statistical comparison among pIIA-C, pT1-2N1a, pT1-2N1b, pT3-4aN1a, pT3-4aN1b, pT4bN1a and pT4bN1b to know whether there were significant differences in CCSS. According to multivariate analysis, the CCSS of patients with pT1-2N1a was similar to that of those with pIIA stage disease (HR, 0.937; 95% CI, 0.838–1.049; *P* = 0.259, using pIIA stage as the reference). Patients with stage pIIB disease had lower 5-year CCSS than those with pT1-2N1a (HR, 0.677; 95% CI, 0.606–0.757; *P* < 0.001, using stage pIIB as the reference) but similar 5-year CCSS to those with pT1-2N1b disease (HR, 0.971; 95% CI, 0.861–1.096; *P* = 0.634). Patients with stage pIIC disease had significantly lower 5-year CCSS than those with pT1-2N1a (HR, 0.254; 95% CI, 0.224–0.287; *P* < 0.001, using stage pIIC as the reference) and those with pT3-4aN1a (HR, 0.601; 95% CI, 0.560–0.645; *P* < 0.001), but higher 5-year CCSS than those with pT4bN1a disease (HR, 1.761; 95% CI, 1.576–1.966; *P* < 0.001) (Table 7).

**Table 7:** Comparison of 5-year CCSS of patients with SLNM and pII stage CC

**Variable**	**HR (95% CI)**	**P**	**HR (95% CI)**	**P**	**HR (95% CI)**	
pTNM stage		<0.001		<0.001		<0.001
IIA	Reference		0.723 (0.688-0.759)	<0.001	0.271 (0.252-0.290)	<0.001
IIB	1.384 (1.317-1.453)	<0.001	Reference		0.374 (0.350-0.401)	<0.001
IIC	3.695 (3.443-3.966)	<0.001	2.671 (2.495-2.859)	<0.001	Reference	
T1-2N1a	0.937 (0.838-1.049)	0.259	0.677 (0.606-0.757)	<0.001	0.254 (0.224-0.287)	<0.001
T3-4aN1a	2.221 (2.109-2.339)	<0.001	1.605 (1.529-1.685)	<0.001	0.601 (0.560-0.645)	<0.001
T4bN1a	6.506 (5.886-7.192)	<0.001	4.703 (4.262-5.189)	<0.001	1.761 (1.576-1.966)	<0.001
T1-2N1b	1.344 (1.189-1.518)	<0.001	0.971 (0.861-1.096)	0.634	0.364 (0.319-0.414)	<0.001
T3-4aN1b	3.060 (2.915-3.211)	<0.001	2.212 (2.115-2.312)	<0.001	0.828 (0.774-0.886)	<0.001
T4bN1b	8.011 (7.328-8.757)	<0.001	5.790 (5.307-6.317)	<0.001	2.168 (1.961-2.397)	<0.001

*P* values refer to comparison between each group and the reference group and were adjusted for year of diagnosis, age, sex, pathological grading, histological type of tumor, and number of LNs dissected as covariates.

## Discussion

LN metastasis is a critical predictor of disease recurrence and CCSS, and therefore an important determinant of postoperative therapy [8]. Various variables, including pathological tumor stage, tumor grade, and degree of differentiation, have been identified as being associated with LN metastases [9,10]. In this study, we found that patients’ age, race, pathological grading, histological type of tumor, pT stage and number of LNs dissected provided risk stratification for patients with LN metastasis. Tumors with solitary positive node always mean more deep tumors and worsen grading than those with negative LNs, and the seventh edition of the AJCC Cancer Staging Manual for colon classified any pT stage with solitary positive node into pIII or pIV, both which means worsen survival outcomes.

Patients with esophageal cancer and SLNM have been considered a distinct prognostic subgroup with cancer outcomes closer to that of patients with node-negative disease and better than any other node-positive subgroup [11]. It has even been suggested that there is no survival difference between patients with SLNM and those with N0 esophageal squamous cell carcinoma; that is, SLNM does not affect the prognosis [12]. Bardia et al. [13] reported that six rectal adenocarcinoma patients with a solitary inguinal LN metastasis survived a mean of 42 months from diagnosis, three of the six patients still being alive after a mean duration of 40 months of follow-up when the article was accepted for publication. It is important to investigate the prognosis of patients with SLNM; the presence of multiple LN metastases is already known to be associated with systematic disease and poor prognosis [14]. However, thus far no studies have investigated the prognosis of CC patients with SLNM.

In this study we analyzed the SEER data of 86,674 CC patients and found significant differences in survival between patients with SLNM and those with pN1b disease, verifying our hypothesis that SLNM is the earliest form of LN invasion and has heterogeneous outcomes. Soni et al. confirmed the sentinel node as the only site of metastasis in 41% of node-positive patients [10] and considered that the patients with SLNM did not have systemic disease. We further investigated survival differences by T stage category and found that patients with SLNM in all five pT stages had a significantly longer 5-year CCSS than did pN1b patients, indicating that CC with a SLNM may have an inherently favorable biologic character.

Of interest is that, in our study, the 5-year CCSS of patients with pT1N1a CC was 92.6%, which is higher than that of those with pIIA (88.4%). The 5-year CCSS of patients with pT1-2N1a stage was similar to that of those with stage pIIA, but significantly greater than that of those with pIIB disease. Patients with pT3-4N1a disease had a better 5-year CCSS than those with pIIC. What could explain why patients with SLNM have a better CCSS than those with no LN metastases? We postulate that the major reasons are incomplete surgical resection and/or inadequate node sampling, resulting in inaccurate TNM staging. In the United States, more than 60% of colon cancer is under-staged after surgery [15]. At least 12 examined LNs is the benchmark for accurately ascertaining pathological node stage. Numerous observational studies of the impact of the number of LNs retrieved in patients with CC have shown a clear survival benefit with increasing numbers of LNs examined, especially in stage II patients [16-18]; our findings are consistent with these data. The more nodes that are examined and found negative, the more likely that a stage II patient is really node-negative, whereas lower nodal counts increase the risk that a node-positive patient will be misclassified as node-negative. When the technique of sentinel lymph node mapping is used, there is a 15% absolute increase in nodal positivity [10]. Such under-staging leads to under-treatment: many under-staged patients do not receive the adjuvant chemotherapy that is essential for survival benefit. About 15% to 20% of stage I/II colon patients develop recurrence within 5 years of diagnosis [19]. The benefits of increased nodal counts in node-positive patients remain controversial. Because we used the number of LNs dissected as a co-variable in our univariate and multivariate survival analyses, our findings suggest that SLNM CC has inherently favorable biologic behavior.

Despite this, patients with positive LNs are routinely referred for adjuvant therapy [20]. NCCN guidelines (version I.2014) recommend adjuvant chemotherapy for stage pIII CC patients, including those with stage pT1-2N1a, but do not recommend adjuvant chemotherapy for stage pII patients who are assessed as low risk. Many physicians assume that pII stage patients have a better CCSS than pIII patients. Also patients with pII stage are less willing to undergo chemotherapy than pIII stage patients in clinical practice [21,22]. Thus, stage pT1-2N1a CC patients may be over-treated and stage pII patients under-treated. Unfortunately, because information about chemotherapy is not available in the SEER database, we were not able to analyze this issue further. Postoperative adjuvant treatment with fluorouracil and levamisole reportedly reduces the mortality rate by more than 30% in patients with stage III CC [23-25]. However, with CCSS as high as 92.6% in patients with pT1N1a stage disease, does adjuvant chemotherapy benefit all patients in this subgroup? AJCC staging was initiated to assess survival and guide clinical practice; we believe it should emphasize the distinctive characteristics of patients with SLNM.

Although this is a large population-based study evaluating the subgroup of CC patients with SLNM, it has several potential limitations. First, the SEER database lacks data concerning several important tumor characteristics (e.g., perineural and lymphovascular invasion), chemotherapy (neoadjuvant and adjuvant), and patient outcome (recurrence and metastasis). Thus, our analyses could not adjust for these potential confounding factors. Second, there may be minor misclassification of pT4 stage. In the first years of this century, the AJCC defined pT4a as CCs infiltrating adjacent organs or structures without perforation of visceral peritoneum and pT4b as those perforating the visceral peritoneum [26]. However, in the 7th AJCC edition, a CC is classified as pT4a when it infiltrates the serosa and as pT4b when it infiltrates adjacent organs: this may influence the classification of pT4a and T4b CCSS. Third, because SEER data provide no information about the distribution of SLNM, we could not tell whether a SLNM was a skip metastasis and therefore could not ascertain whether there is a difference in survival between skip and no skip groups.

## Conclusion

In conclusion, our study shows that patients with SLNM have a better 5-year CCSS than patients with pN1b disease. Patients with pT1-2N1a stage and those with p IIA have a similar 5-year CCSS. Patients with pT3-4aN1a stage have a higher 5-year CCSS rate than those with pIIC disease. The overwhelming advantage in long-term survival of CC patients with SLNM over those with pN1b stage warrants careful attention in clinical practice and TNM stage revision.

## Abbreviations

AJCC: American Joint Committee on Cancer; CCSS: colorectal cancer cause-specific survival rate; CRC: colorectal cancer; CC: colon cancer; LN: lymph node; NCCN: National Comprehensive Cancer Network; SEER: National Cancer Institute’s Surveillance, Epidemiology, and End Results; SLNM: solitary lymph node metastasis.

## Competing interests

The authors declare that they have no competing interests.

## Authors’ contributions

QGL and SJC designed the study. YWW and DWL provided the databases. QGL, YWW, GXC and SJC assembled and analyzed the data. QGL, GXC and YWW wrote the manuscript. All authors read and approved the final manuscript.

## Pre-publication history

The pre-publication history for this paper can be accessed here:

http://www.biomedcentral.com/1471-2407/14/368/prepub
